# High Urban-Rural Inequities of Abdominal Obesity in Malawi: Insights from the 2009 and 2017 Malawi Noncommunicable Disease Risk Factors Surveys

**DOI:** 10.3390/ijerph191911863

**Published:** 2022-09-20

**Authors:** Sékou Samadoulougou, Mariam Diallo, Kadari Cissé, Calypse Ngwasiri, Leopold Ndemnge Aminde, Fati Kirakoya-Samadoulogou

**Affiliations:** 1 Evaluation Platform on Obesity Prevention, Quebec Heart and Lung Institute, Quebec, QC G1V 4G5, Canada; 2 Centre for Research on Planning and Development, Université Laval, Quebec, QC G1V 0A6, Canada; 3 Département de Biochimie et Microbiologie, UFR-SVT, Université Joseph Ki-Zerbo, Ouagadougou 03 BP 7021, Burkina Faso; 4 Centre de Recherche en Epidémiologie, Biostatistique et Recherche Clinique, Ecole de Santé Publique, Université Libre de Bruxelles, Route de Lennik, 808, 1070 Brussels, Belgium; 5 School of Medicine and Dentistry, Griffith University, Gold Coast, QLD 4215, Australia

**Keywords:** abdominal obesity, urban, rural, change, noncommunicable diseases, Malawi

## Abstract

Geographical disparities in abdominal obesity (AO) exist in low-income countries due to major demographic and structural changes in urban and rural areas. We aimed to investigate differences in the urban–rural prevalence of AO in the Malawi population between 2009 and 2017. We conducted a secondary analysis of data from the Malawi 2009 and 2017 STEPS surveys. AO (primary outcome) and very high waist circumference (secondary outcome) were defined using WHO criteria. Prevalence estimates of AO and very high waist circumference (WC) were standardized by age and sex using the age and sex structure of the adult population in Malawi provided by the 2018 census. A modified Poisson regression analysis adjusted for sociodemographic covariates was performed to compare the outcomes between the two groups (urban versus rural). In total, 4708 adults in 2009 and 3054 adults in 2017 aged 25–64 were included in the study. In 2009, the age–sex standardized prevalence of AO was higher in urban than rural areas (40.9% vs 22.0%; adjusted prevalence ratio [aPR], 1.51; 95% confidence interval [CI], 1.36–1.67; *p* < 0.001). There was no significant trend for closing this gap in 2017 (urban 37.0% and rural 21.4%; aPR, 1.48; 95% CI, 1.23–1.77; *p* < 0.001). This urban–rural gap remained and was slightly wider when considering the ‘very high WC’ threshold in 2009 (17.0% vs. 7.1%; aPR, 1.98; 95%CI, 1.58–2.47; *p* < 0.001); and in 2017 (21.4% vs. 8.3%; aPR, 2.03; 95%CI, 1.56–2.62; *p* < 0.001). Significant urban–rural differences exist in the prevalence of AO and very high WC in Malawi, and the gap has not improved over the last eight years. More effective weight management strategies should be promoted to reduce health care disparities in Malawi, particularly in urban areas.

## 1. Introduction

Overweight and obesity are important determinants of noncommunicable diseases (NCDs), especially cardiovascular diseases (CVDs), diabetes, musculoskeletal disorders, and some cancers [1]. An even greater risk is associated with abdominal or visceral obesity, which is the biggest driver of obesity-related CVD [2]. Globally, over 650 million adults were obese in 2016 after the prevalence of obesity doubled between 1980 and 2015 in 73 countries while continuing to increase in others [1,3]. Sub-Saharan Africa (SSA) has the highest rising prevalence of overweight and obesity at about 49.6% in some countries, compared to 31.3% elsewhere around the world [1].

Urbanization is frequently mentioned as the most important contributor to the rising prevalence of overweight and obesity, owing to the higher access to unhealthy meals and sedentary lifestyles in urban settings [4,5,6]. More recently, rising body-mass index (BMI) levels in rural areas was shown to be the main driver of global obesity epidemics in adults, while the contribution from rural to urban migration was small [7]. However, the reliance on BMI alone is insufficient to fully assess and mitigate the health-related risks of obesity. Waist circumference (used for defining abdominal obesity) has been consistently shown to be superior to BMI in predicting CVD risk and alone is a critical factor that can be used to measure the reduction of obesity-related CVD risk [2,8].

Given the strong association between abdominal obesity and CVDs, monitoring its prevalence and time trends is essential to measure progress in reducing NCD risk and identifying high-risk populations. Studies on the prevalence and time trends of abdominal obesity have been conducted across different populations worldwide [9,10,11,12,13,14,15,16]. However, data from SSA countries is limited, and those that exist are most often localized (cities, regions, and villages) and do not necessarily reflect the national situation [17].

In Malawi, about 1 in 4 adults (28.8%) had abdominal obesity in 2009 [18], and overweight and obesity were found to be generally high in both rural and urban areas [19]. Furthermore, Malawian adult women were found to have greater adiposity and more adverse lipid profiles compared to the men [20]. The prevention and control of NCDs are included in Malawi’s Health Sector Strategic Plan II (HSSP II) [21], which is in line with the World Health Organization’s Global Action Plan for the prevention and control of NCDs [22]. The HSSP II contributes to the Sustainable Development Goals via interventions in the basic health package and action on social determinants of health [21]. Regarding nutrition, the Malawi National Multi-Sector Nutrition Policy (NMNP) 2018–2022 [23], implemented through the National Nutrition Strategic Plan 2018–2022, works towards preventing and treating nutrition-related NCDs. Significant progress was achieved with the adoption of national nutrition policies and strategic plans. Establishing a multi-sectoral coordinating institution, expanding coverage of evidence-based, high-impact interventions, providing a framework for standardization and improvement of nutrition service delivery, and placing nutrition high on the National Development Agenda are all notable positive achievements [23].

Despite these policy actions, there is limited national evidence for the temporal changes in abdominal obesity and rural-urban disparities that could potentially indicate the effectiveness of these policies. Rapid urbanization in SSA combined with changing lifestyles (even in rural areas) may explain the reduction in the disparities of abdominal obesity between urban and rural areas [24]. Focus has mostly been on the difference in the prevalence of abdominal obesity among rural and urban populations at a particular time point [25], whereas studies comparing trends in the prevalence of abdominal obesity between urban and rural areas are scarce. In this context, our study aimed to assess the trends in the prevalence of abdominal obesity among urban and rural adult populations in Malawi between 2009 and 2017. We anticipate that the results will contribute to shaping the implementation of the national NCD policy in Malawi and provide a basis for measuring the effectiveness of government interventions on the dynamics of abdominal obesity in urban and rural areas.

## 2. Materials and Methods

### 2.1. Study Type

We performed a secondary analysis of two nationwide population-based cross-sectional surveys conducted in 2009 and 2017 following the WHO STEPwise approach to NCD risk factor surveillance in Malawi. Malawi is a landlocked country located in southeastern Africa which is bordered by Mozambique to the south, southwest, and southeast, Zambia to the northwest, and Tanzania to the northeast.

### 2.2. Study Population 

In 2009, the survey involved 5206 participants aged 25 to 64 years with an overall response rate of 95.5%. The 2017 survey involved 4187 participants aged 18 to 69 with an overall response rate of 99.5%. Subjects with missing data on waist circumference, BMI, pregnant women, and participants under 25 years of age or older than 64 years (in 2017, for reasons of comparability), were removed from the analysis. 

### 2.3. Data Collection

The data were collected using a multi-stage cluster sampling design. In the first stage, enumeration areas were selected in both urban and rural areas based on the list of enumeration areas (EAs) from the June 2008 census of population and dwellings. For both surveys, EAs were randomly selected using size-related sampling. In each EA, households were selected by systematic sampling. Only one eligible participant was selected at the household level using the Kish sampling method [26]. Households without an eligible participant were not replaced. This sampling method was recommended by the WHO STEPwise methodology to reduce bias in the survey [26].

Data collection was done in three stages using personal digital assistants (PDAs) [26]. The first step involved collecting sociodemographic and behavioral information (smoking, alcohol consumption, diet, physical activity), while the second step involved the collection of anthropometric and blood pressure measurements. In the third and final step, biochemical measurements (assessment of blood sugar and cholesterol levels) were done.

In each household, the data collection took 2 days. On the first day, a questionnaire was used to collect data on the sociodemographic and behavioral characteristics of participants, followed by physical measurements (anthropometric and blood pressure measurements). On the second day, biochemical parameters were collected. 

Waist circumference was measured using a ribbon graduated in centimeters. The measurements were taken in the mid-axillary line halfway between the last rib and the upper iliac crest. The values were taken to the nearest 0.1 cm. A ribbon placed horizontally at the point of maximum circumference above the buttocks was used to measure the hip circumference. 

### 2.4. Study Variables

#### 2.4.1. Dependent Variable

The variables of interest (AO and very high WC) were defined based on WHO criteria [27]. Men with a WC ≥ 94 cm and women with a WC ≥ 80 cm were considered as having AO. Similarly, men with a WC ≥ 102 cm and women with a WC ≥ 88 cm were considered as having very high WC. In 2011, the WHO recognized that the risk of metabolic complications is “substantially” elevated at this ‘very high WC’ threshold, given that at this threshold people typically require an intervention to reduce their weight [28,29]. This secondary outcome was used to estimate the proportion of adults who needed to reduce their weight [29]. The dependent variable was categorized as “1” for the presence of abdominal obesity and “0” for absence.

#### 2.4.2. Adjustment Variables

The urban and rural disparity in the prevalence of abdominal obesity was adjusted for the following variables: gender (male, female), age (25–34, 35–44, 45–54, and 55–64), marital status (married/cohabiting or single/separated/divorced/widowed), and level of education (uneducated, primary, secondary/tertiary).

### 2.5. Statistical Analyses

We described the characteristics of the study populations considering the survey weights and the sampling design. Abdominal obesity was described as proportions, and the prevalence of abdominal obesity and very high WC were standardized for age and sex using the age and sex structure of the Malawian adult population in 2018 (Census of 2018). Differences between 2009 and 2017, as well as between rural-urban for each outcome, were estimated by looking at the overlap of the 95% confidence intervals in sociodemographic subgroups. A modified Poisson regression using a generalized estimating equation was performed to determine the disparity between urban and rural residents regarding abdominal obesity in 2009 and 2017. The model was adjusted for sociodemographic characteristics of study participants, and the prevalence ratio (PR) and the confidence interval were reported. Differences were considered statistically significant for a threshold of 5% (*p* < 0.05). The data were analyzed using STATA software (Statacorp LLC, College Station, TX, USA) version 17.0.

## 3. Results

**Figure 1:**
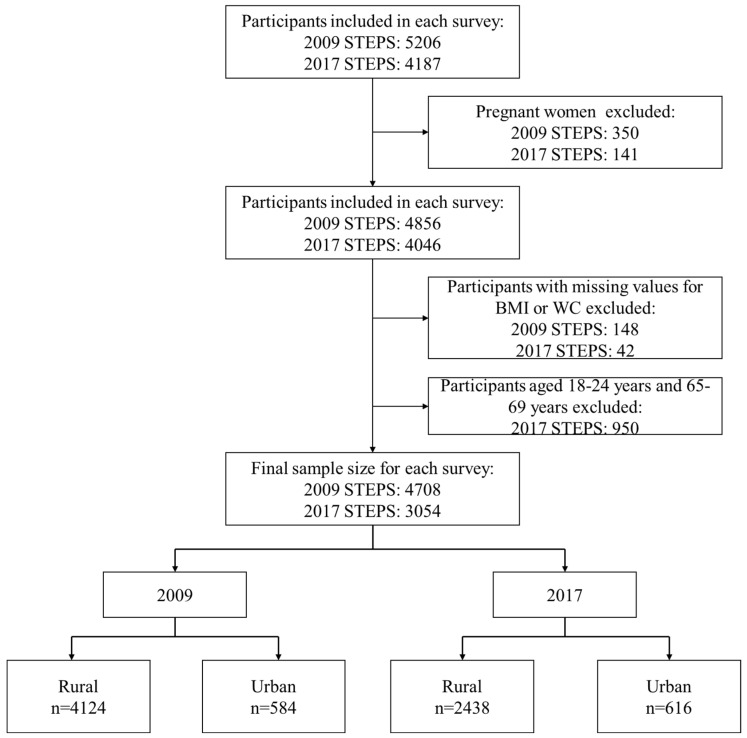
Flowchart of study participants.

A total of 4708 adults in 2009 (4124 in rural areas and 584 in urban areas) and 3054 adults in 2017 (2438 in rural areas and 616 in urban areas) aged 25–64 years were included in our analysis. Figure 1 shows the process used to obtain our final study population.

### 3.1. Sociodemographic Characteristics of Study Participants in Malawi in 2009 and 2017

In the urban areas, nearly two-thirds of the participants were females (58.7% in 2009 and 61.7% in 2017), whereas, in the rural regions, just over half the participants were males (51.9% in 2009 and 50.4% in 2017). (Table 1).

### 3.2. Prevalence of Abdominal Obesity and Proportion of Adults Requiring Weight Management

**Figure 2:**
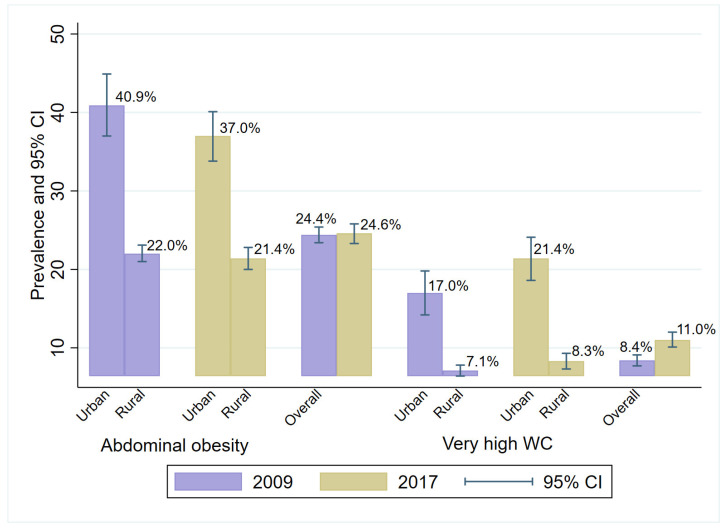
Age and sex-standardized prevalence of abdominal obesity and of adults requiring weight management in Malawi in 2009 and 2017.

In both study periods, the prevalence of AO was higher in urban compared to rural areas (40.9% vs. 22.0% in 2009 and 37.0% vs 21.4% in 2017, respectively) and the gap was wider in 2009 (Figure 2). The overall prevalence of AO was 24.4% in 2009 and 24.6% in 2017 (Table 2 and Figure 2). A similar pattern was observed for very high WC, with a higher prevalence in the urban areas (17.0% vs. 7.1% in 2009 and 21.4% vs 8.3% in 2017, respectively). The urban–rural gap was wider in 2017 compared to 2009 (Figure 2). In comparison to 2009 (8.4%; 95%CI: 7.7–9.1), the prevalence of very high WC was statistically wider in 2017 (11.1%; 95%CI: 10.1–12.0) (Table 3). 

Urban women, respondents aged 35 years and older, and people with higher levels of education had a higher prevalence of AO compared to their rural counterparts across both study years (Table 2). After adjusting for sociodemographic covariates, the prevalence of AO was about 1.51 times higher among urban residents than rural residents in 2017 (aPR: 1.51, 95%CI: 1.36–1.67), and 1.48 times higher among urban residents compared to rural residents in 2009 (aPR: 1.48, 95%CI: 1.23–1.77) (Table 4).

The prevalence of very high WC was also significantly higher in the urban areas compared to the rural areas and increased from 2009 to 2017. In 2009, very high WC was 1.98 times higher in urban areas (aPR: 1.98, 95%CI: 1.58–2.47), whereas in 2017, very high WC was 2.03 times higher in urban than rural areas (aPR: 2.03, 95%CI: 1.56–2.62) (Figure 2 and Table 4). 

There was no significant trend for closing this gap in 2017 (urban 37.0% and rural 21.4%) (Figure 2). More females than males had a higher prevalence of AO, especially among urban respondents. Older respondents had higher prevalence of AO than younger respondents. There was a gradient in high prevalence of AO by education (Table 2). This prevalence was 1.48 times higher among urban residents compared to rural residents (aPR: 1.48, 95% CI: 1.23–1.77) after adjustment for sociodemographic covariates (Table 4). 

## 4. Discussion

### 4.1. Key Results

This study reports, for the first time, the urban–rural disparities in the prevalence of abdominal obesity and its trend among the adult population in Malawi. Abdominal obesity and very high WC prevalence were higher among urban residents than rural residents in 2009 and in 2017. This study showed a high and stagnant prevalence of abdominal obesity in the adult population between 2009 and 2017. By contrast, the proportion of the population with very high WC increased between 2009 and 2017.

### 4.2. Trend in Urban and Rural Disparities of Abdominal Obesity

Studies that have analyzed the trends in the prevalence of abdominal obesity are rare in Africa. Nevertheless, recent studies at the global level have shown an increasing prevalence of abdominal obesity. Wong et al. [16] noted an increased prevalence of abdominal obesity among the world adult population in recent decades, from 31.3% between 1985–1999 to 48.3% between 2010–2014. In China, Shen et al. [24] have noted that the urban and rural disparities in the prevalence of abdominal obesity have changed from 1993 to 2011. The prevalence of abdominal obesity increased in both urban and rural areas during their study period, with a more rapid increase in rural areas. This result was attributed to the changes in China’s social and economic structures led by urbanization [24]. In our study, we found an increased prevalence proportion of very high WC, particularly in urban areas, suggesting that the gap between urban and rural areas is expanding. 

The global prevalence of abdominal obesity reported by Wong et al. [16] is much higher than in our study, and the trends over the years seem to be higher compared to our study. Globally, the growing trend of abdominal obesity is due to economic growth and urbanization, particularly in low- and middle-income countries. As shown by Wong et al. [16], the global prevalence of abdominal obesity was 41.5%, and this prevalence is higher in high-income countries (44.7%) than in low-income countries (30.6%). However, the consequences of abdominal obesity on lipid profile seem worse in low-income countries like Malawi [20]. This difference could be explained by the level of economic development between these regions of the world. In this sense, studies have revealed that lifestyle change (consumption of high-energy foods, lack of physical activity, and sedentary behavioral patterns) could be attributable to scientific and technological progress [30,31], which is associated with obesity. An unhealthy diet (very high in calories and low in dietary fiber) and sedentary behaviors (lack of vigorous physical activity, prolonged computer and car use) [32,33] are known to increase the risk of abdominal obesity significantly. 

The trends in the prevalence of abdominal obesity are not well documented in SSA. Despite the limited trend data, studies in several SSA countries have also shown a high prevalence of abdominal obesity. In 2021, Cissé et al. showed that the prevalence of abdominal obesity in Burkina Faso was 22.5%, which was lower than the 28.8% reported by Msyamboza et al. in Malawi [18]. In Nigeria, Olatunbosum et al. estimated the prevalence of abdominal obesity at 30.1% in urban areas [34]. According to the International Diabetes Federation threshold, the prevalence of abdominal obesity in Togo was 48.8% [35], 52% in Kenya [29], and 67% in South Africa [36]. The prevalence of very high abdominal obesity (persons requiring a weight loss plan) was 10.2% in Burkina Faso [37], 11.8% in Uganda [38], and 33.7% in Togo [35]. While the prevalence in Burkina Faso and Uganda is on par with our findings, there is a difference with the estimates from Togo. In general, the prevalence of abdominal obesity in SSA is gradually approaching that of high-income countries because most of these countries, including Malawi, are undergoing a rapid epidemiological, nutrition, and economic transition. This is likely to exacerbate health deficits in these countries [35]. Indeed, they will have to deal with an expanding double burden of communicable and noncommunicable diseases. Gouda et al. [39] estimated that the increase in NCDs in SSA will soon outweigh the communicable disease burden. In their 2017 analysis, they reported that the disability-adjusted life years (DALYs) attributable to NCDs in 2017 were almost equivalent to DALYs due to infectious, maternal, neonatal, and nutritional diseases combined in SSA. 

Abdominal obesity is a component of metabolic syndrome. The latter influences the pathogenesis of CVD, diabetes, and some cancers via the stimulation of factors mediating insulin resistance, systematic inflammation, and dyslipidemia in people with normal weight. This risk is even higher with increased abdominal fat [40,41]. The African adult population does not have this perception of abdominal obesity and rather view it as a sign of wealth and opulence. In Sub-Saharan Africa, a preference for larger body size and its association with health and wellbeing is well known and documented [42,43]. The government of Malawi needs to implement awareness-raising campaigns, early detection, and strategies to promote weight loss to reduce the risk of abdominal obesity and prevent a future epidemic of cardiometabolic complications [37].

We also found that the expanding gap between urban and rural areas is most pronounced among women. In Cameroon, a study conducted among students revealed that while the prevalence of abdominal obesity remained unchanged among men, it increased from 6.5% in 2009 to 11.7% in 2014 among women [44]. Many studies have reported a higher prevalence of abdominal obesity in women than men. Indeed, Msyamboza et al. [18] noted that in Malawi, the prevalence of abdominal obesity was 52.8% among women versus 5% among men. In the African socio-cultural context, overweight/obesity is a sign of beauty, respect, dignity, and trust [18,45,46]. In addition, behavioral factors (physical inactivity), genetic, and biological factors are considered in the female predisposition to develop abdominal obesity. Biologically, gestation has been reported to lead to increased WC via increased visceral and abdominal fat after childbirth [47]. The difference in the proportion of abdominal obesity according to sex is also related to the secretion of sex steroid hormones, especially during adolescence, when they cause a divergence in the bodily structure and composition between men and women [38]. In addition, after menopause, body fat is redistributed to the abdominal area [48], which explains the high prevalence of abdominal obesity in women over 45 years. There is a need for efforts to change the socio-cultural perception of obesity in SSA in general and in Malawi and to establish comprehensive abdominal and general obesity prevention and control programs that account for gender specificities.

We also found that older urban respondents with high levels of education had a high prevalence of AO and very high WC compared to the rural respondents. These findings have also been reported in Burkina Faso [37], Togo [35], Uganda [38], and in Benin [25]. Ageing is known to be related to abdominal obesity, owing to a myriad of metabolic and physiologic changes [41,49]. In addition, educational level is positively correlated with income, both of which are associated with obesity, particularly in urban settings with more motorized travel, sedentary behaviors, and the proliferation and consumption of energy-dense foods [50]. Our findings indicate that, like other Sub-Saharan African and South Asian countries, Malawi is in the early phases (stage 1) of the obesity transition where obesity is higher in those with high socioeconomic status compared to lower socioeconomic and rural dwelling counterparts [51]. 

### 4.3. Implication

We can infer from our findings that national efforts to reduce AO need to consider some focus on key population groups, such as urban women and those with higher socio-economic status. At a primary level, health professionals need to include the diagnosis and management of abdominal obesity in their routine practice to reduce the burden of this risk factor. The practice of sports and a healthy diet should be a leitmotiv at the individual level and within families. This tripartite (government, health professionals, and community) intervention is necessary to effectively reduce the burden of noncommunicable diseases in Malawi.

However, effectively addressing the burden of abdominal and general obesity in Malawi would require a shift from an individual-only perspective (such as the adoption of a healthy lifestyle and motivation to participate in sports) to a more systemic and whole of population perspective, which should sometimes include mandatory and regulatory policies. 

### 4.4. Limitations and Strengths

As our study is a secondary analysis of existing cross-sectional data, the study design makes it improbable for us to tease out the causal inferences. In addition, to ensure accurate measurement estimates, it is recommended to average the same process repeated two to three times. However, WC, weight, and height measurements were done only once in this study, which may make our estimates liable to measurement error. Another limitation is that the data from 2017 may no longer reflect the current prevalence of abdominal obesity, especially in Malawi where industrialization is in progress.

Despite these potential shortcomings, our study has noteworthy strengths. To our knowledge, it provides, for the first time in Malawi, the rural-urban disparities in the evolution of abdominal obesity over almost a decade, a significant addition to the limited evidence in SSA. Another key strength of this study is the use of a large sample of nationally representative adults in Malawi using the standardized WHO STEPS methodology.

## 5. Conclusions

Our study revealed high urban–rural inequities in the prevalence of abdominal obesity despite the implementation of the national NCD plan since 2013 in Malawi. The increased trend in urban areas was mainly driven by the increased prevalence of very high WC among women. In Malawi, where industrialization is in progress, it is essential to improve weight-loss strategies, particularly targeting women in urban areas.

## Figures and Tables

**Table 1:** Characteristics of rural and urban Malawian adults in 2009 and 2017.

Characteristics	2009			2017			Total		
	Rural (n = 4124)	Urban (n = 584)	*p* Value	Rural (n = 2438)	Urban (n = 616)	*p* Value	2009	2017	*p* Value
Sex			<0.001			0.004			0.006
Men	51.9	41.3		50.4	38.3		50.7	49.0	
Women	48.1	58.7		49.6	61.7		49.3	51.0	
Age (years)			<0.001			0.14			<0.001
25–34	43.6	53.2		40.1	46.9		44.7	40.8	
35–44	25.5	23.6		32.9	28.7		25.3	32.4	
45–54	18.6	12.6		16.9	16.3		17.9	16.8	
55–64	12.3	10.6		10.2	8.1		12.1	9.9	
Marital status			0.25			<0.001			<0.001
Single	24.3	21.4		23.5	34.6		23.9	24.7	
Married	75.7	78.6		76.5	65.4		76.1	75.3	
Level of education			<0.001			<0.001			<0.001
No Schooling	22.8	13.4		12.6	6.1		21.7	11.9	
Primary	64.0	49.8		68.6	43.1		62.4	65.8	
Secondary/higher	13.2	36.8		18.8	50.8		15.9	22.4	
Profession			0.001			0.031			0.11
Unemployed	49.1	60.9		43.4	53.2		49.6	55.5	
Employed	50.9	39.1		56.6	46.8		50.4	44.5	

**Table 2:** Age and sex-standardized prevalence of abdominal obesity by sociodemographic characteristics of adults in Malawi, 2009–2017.

Characteristics *	2009		2017		Total	
	Rural	Urban	Rural	Urban	2009	2017
All					24.4 (23.4–25.4)	24.6 (23.3–25.8)
Sex						
Men	2.3 (1.5–3.1)	18.1 (11.6–24.5)	2.7 (1.5–3.4)	11.8 (7.6–16.0)	3.6 (2.7–4.5)	4.1 (3.0–5.2)
Women	40.4 (38.5–42.3)	62.0 (57.3–66.7)	39.2 (36.6–41.7)	60.3 (55.7–64.9)	43.7 (41.9–45.5)	43.7 (41.5–45.9)
Age						
25–34	18.3 (16.8–19.9)	34.1 (29.8–38.4)	19.2 (17.0–21.4)	28.5 (24.0–32.9)	20.9 (19.5–22.4)	21.4 (19.4–23.3)
35–44	23.1 (21.1–25.2)	49.0 (40.3–57.8)	21.5 (19.0–24.1)	37.5 (31.3–43.7)	26.0 (23.9–28.0)	24.8 (22.4–27.1)
45–54	27.6 (25.0–30.2)	43.4 (31.8–54.9)	25.3 (22.2–28.4)	48.4 (40.6–56.2)	28.9 (26.3–31.4)	30.2 (27.2–33.2)
55–64	25.4 (22.5–28.3)	39.7 (28.4–51.2)	24.5 (20.7–28.4)	52.0 (42.3–61.7)	26.9 (24.0–29.7)	28.8 (25.0–32.6)
Marital status						
Single	20.8 (18.7–22.8)	41.8 (28.8–54.7)	18.9 (16.6–21.2)	34.0 (28.4–39.7)	22.7 (20.6–24.9)	22.1 (19.9–24.3)
Married or cohabiting	22.6 (21.3–23.9)	41.6 (37.2–45.9)	23.0 (21.1–24.7)	38.1 (34.1–42.1)	25.2 (23.9–26.4)	26.0 (24.4–27.6)
Level of education						
No schooling	21.3 (19.3–23.2)	32.7 (23.7–41.7)	21.3 (17.1–25.5)	30.4 (21.9–38.8)	22.1 (20.1–24.0)	22.9 (19.0–26.7)
Primary school	22.6 (21.2–23.9)	35.1 (30.2–40.0)	20.8 (19.1–22.5)	33.1 (28.7–37.5)	24.0 (22.7–25.3)	22.7 521.1–24.3)
Secondary/higher school	26.2 (21.3–31.1)	57.0 (50.3–63.7)	29.0 (24.3–33.6)	42.2 (37.7–46.7)	36.0 (32.2–39.8)	35.1 (32.2–38.0)
Profession						
Unemployed	22.9 (21.1–24.6)	42.1 (37.1–47.0)	22.9 (20.3–25.4)	39.2 (34.9–43.5)	25.7 (24.1–27.3)	27.4 (25.2–29.6)
Employed	21.4 (20.1–22.8)	35.0 (30.2–39.8)	20.4 (18.7–22.1)	33.2 (28.3–38.2)	23.1 (21.9–24.4)	22.4 (20.8–24.0)

* Data are presented as percentage with 95% confidence interval.

**Table 3:** Age and sex standardized prevalence of very high waist circumference by socio-demographic characteristics of adults in Malawi, 2009–2017.

Characteristics *	2009		2017		Total	
	Rural	Urban	Rural	Urban	2009	2017
All					8.4 (7.7–9.1)	11.0 (10.1–12.0)
Sex						
Men	0.6 (0.2–1.0)	4.5 (1.0–7.9)	0.4 (0.1–0.9)	4.9 (2.0–7.7)	0.9 (0.5–1.4)	1.2 (0.6–1.8)
Women	13.2 (11.9–14.5)	28.6 (24.1–33.0)	15.6 (13.7–17.4)	36.8 (32.2–41.3)	15.4 (14.1–16.6)	20.2 (18.4–22.0)
Age						
25–34	4.7 (3.7–5.5)	12.2 (9.4–15.1)	6.3 (4.8–7.7)	15.3 (11.7–18.9)	6.0 (5.1–6.9)	8.5 (7.1–9.9)
35–44	7.9 (6.4–9.3)	16.9 (11.6–22.2)	9.8 (7.8–11.7)	22.8 (17.2–28.3)	9.1 (7.7–10.5)	12.5 (10.6–14.4)
45–54	10.3 (8.4–12.2)	27.1 (16.6–37.6)	10.7 (8.2–13.1)	27.5 (20.9–34.0)	11.7 (9.7–13.6)	14.3 (11.8–16.7)
55–64	10.1 (7.9–12.3)	20.4 (11.7–29.2)	8.6 (6.0–11.2)	33.0 (23.1–42.9)	11.2 (9.1–13.3)	12.3 (9.5–15.2)
Marital status						
Single	5.4 (4.4–6.4)	10.8 (6.8–14.8)	7.0 (5.4–8.5)	22.0 (16.9–27.2)	6.0 (5.0–7.0)	10.1 (8.5–11.7)
Married	8.0 (7.1–8.9)	19.1 (15.6–22.5)	9.2 (7.9–10.5)	20.8 (17.3–24.3)	9.5 (8.6–10.4)	11.6 (10.3–12.8)
Level of education						
No Schooling	6.0 (4.8–7.2)	13.5 (7.6–19.4)	5.9 (3.4–8.8.3)	18.8 (10.6–27.1)	6.6 (5.4–7.8)	8.1 (5.4–10.8)
Primary School	7.5 (6.6–8.4)	14.4 (11.1–17.7)	7.7 (6.5–8.9)	18.5 (14.6–22.4)	8.3 (7.5–9.2)	9.4 (8.2–10.5)
Secondary/higher School	8.7 (4.8–12.5)	28.1 (21.6–34.5)	17.9 (13.7–22.2)	26.1 (21.8–30.4)	15.9 (12.3–19.6)	21.1 (18.3–24.0)
Profession						
Unemployed	8.1 (6.8–9.2)	19.1 (15.1–23.0)	8.9 (7.1–10.7)	22.9 (18.9–26.8)	9.8 (8.6–11.0)	12.9 (11.2–14.7)
Employed	6.5 (5.6–7.4)	13.5 (10.1–16.9)	8.0 (6.7–9.1)	20.9 (16.6–25.1)	7.4 (6.5–8.2)	10.0 (8.8–11.2)

* Data are presentented as percentage with 95% confidence interval.

**Table 4:** Abdominal obesity and very high WC according to urban/rural residence in 2009 and 2017.

Variable	2009				2017			
	cPR (95% CI)	*p*-Value	aPR * (95% CI)	*p*-Value	cPR (95% CI)	*p*-Value	aPR * (95% CI)	*p*-Value
Primary outcome								
Residence		<0.001		<0.001		<0.001		<0.001
Rural	1		1		1		1	
Urban	1.84 (1.61–2.09)		1.51 (1.36–1.67)		1.79 (1.51–2.12)		1.48 (1.23–1.77)	
Secondary outcome								
Residence		<0.001		<0.001		<0.001		<0.001
Rural	1		1		1		1	
Urban	2.40 (1.87–3.07)		1.98 (1.58–2.47)		2.72 (2.11–3.48)		2.03 (1.56–2.62)	

cPR: crude prevalence ratio; aPR: adjusted prevalence ratio, * results were adjusted for sex, age, level of education, and marital status.

## Data Availability

The data used in this study is publicly available upon request to the WHO STEPS program (https://extranet.who.int/ncdsmicrodata/index.php/catalog, accessed on 5 August 2022).
